# Tumor Microenvironment Features and Chemoresistance in Pancreatic Ductal Adenocarcinoma: Insights into Targeting Physicochemical Barriers and Metabolism as Therapeutic Approaches

**DOI:** 10.3390/cancers13236135

**Published:** 2021-12-06

**Authors:** Tiago M. A. Carvalho, Daria Di Molfetta, Maria Raffaella Greco, Tomas Koltai, Khalid O. Alfarouk, Stephan J. Reshkin, Rosa A. Cardone

**Affiliations:** 1 Department of Biosciences, Biotechnologies and Biopharmaceutics, University of Bari, 70126 Bari, Italy; daria.dimolfetta@uniba.it (D.D.M.); grecoraffaella@hotmail.it (M.R.G.); stephanjoel.reshkin@uniba.it (S.J.R.); rosaangela.cardone@uniba.it (R.A.C.); 2 Via Pier Capponi 6, 50132 Florence, Italy; tkoltai@hotmail.com; 3 Al-Ghad International College for Applied Medical Sciences, Al-Madinah Al-Munwarah 42316, Saudi Arabia; Alfarouk@hala-alfarouk.org

**Keywords:** pancreatic ductal adenocarcinoma, tumor microenvironment, chemoresistance, treatment, extracellular matrix, desmoplasia, acidic pH, hypoxia, metabolism

## Abstract

**Simple Summary:**

Pancreatic ductal adenocarcinoma (PDAC) has an extremely poor prognosis. The lack of early diagnosis and the absence of suitable biomarkers coupled with resistance to available therapeutic options has made PDAC one of the deadliest cancers. Despite advances in diagnostics and therapeutics, the prognosis of PDAC remains dismal. PDAC has a prominent desmoplastic stromal microenvironment that includes a dense extracellular matrix together with a series of activated cell types, hypoxia, and an acidic extracellular pH. This activated desmoplastic stroma compromises treatments yet, despite the recognition of its importance, it has not been comprehensively studied in this role. Moreover, PDAC metabolic reprogramming has also been found to be one of the key factors involved in treatment failure. Here, we critically review the role of the various stromal components in determining resistance to available therapeutics with the hope that its comprehensive understanding, if employed in the appropriate combination therapy, may make this recalcitrant cancer more manageable.

**Abstract:**

Currently, the median overall survival of PDAC patients rarely exceeds 1 year and has an overall 5-year survival rate of about 9%. These numbers are anticipated to worsen in the future due to the lack of understanding of the factors involved in its strong chemoresistance. Chemotherapy remains the only treatment option for most PDAC patients; however, the available therapeutic strategies are insufficient. The factors involved in chemoresistance include the development of a desmoplastic stroma which reprograms cellular metabolism, and both contribute to an impaired response to therapy. PDAC stroma is composed of immune cells, endothelial cells, and cancer-associated fibroblasts embedded in a prominent, dense extracellular matrix associated with areas of hypoxia and acidic extracellular pH. While multiple gene mutations are involved in PDAC initiation, this desmoplastic stroma plays an important role in driving progression, metastasis, and chemoresistance. Elucidating the mechanisms underlying PDAC resistance are a prerequisite for designing novel approaches to increase patient survival. In this review, we provide an overview of the stromal features and how they contribute to the chemoresistance in PDAC treatment. By highlighting new paradigms in the role of the stromal compartment in PDAC therapy, we hope to stimulate new concepts aimed at improving patient outcomes.

## 1. Introduction

Pancreatic ductal adenocarcinoma (PDAC) is the fourth leading cause of cancer-related mortality in western countries and is projected to be the second-leading cause of cancer-related death in the United States by 2030 [1]. Despite significant breakthroughs in cancer research, PDAC remains a malignant disease with a high mortality. It is among the most chemoresistant cancers due to the broad heterogeneity of the genetic mutations and the dense stromal environment [2]. Due to the lack of both early diagnostic strategies and premature symptoms before the disease reaches its advanced stage, approximately 85% of tumors are not resectable at the time of diagnosis [3], making the median patient survival only 6–10 months [4]. Although several chemotherapies have reported some benefits, they are not enough to prolong survival and improve a patient’s quality of life [5]. Despite improvements made in the approaches for detecting and managing pancreatic cancer, the five-year survival rate only reached 9% in 2020 [6]. A prominent feature of the PDAC microenvironment is an extensive desmoplasia that consists of a highly fibrotic and stiff extracellular matrix (ECM) principally composed of collagen I, fibronectin and hyaluronan, which are secreted by alpha-muscle actin-positive fibroblasts (also known as myofibroblasts or activated pancreatic stellate cells (PSCs)) [7]. Modifications that the ECM architecture suffers during cancer progression have been deeply explored, since it has been recognized that atypical ECM architecture influences therapeutic outcomes specifically by modulating (i) tumor biomechanics [8]; (ii) cancer cell migration/invasion [9,10,11]; and (iii) drug penetration into the tumor [12]. The existence of this dense tumor microenvironment (TME) may be the main reason that therapies targeting specifically only cancer-associated molecular pathways have not given satisfactory results [13].

The TME was first proposed by Ioannides in 1993, and referred especially to the local environment where tumors occurred and developed [14]. Commonly, the TME of PDAC is characterized by abundant stroma, hypoxia, a deficient blood supply and elevated immunosuppression [15]. Studies have shown that the TME, including cancer-related fibroblasts (CAFs), stellate cells, diverse immune cells and cytokines released by them, are involved in the control of the proliferation, metastasis, chemoresistance and immunotherapy of pancreatic cancer cells [16]. Factors associated with TME such as cell plasticity, heterogeneity of the tumor, composition of the tumor stroma, epithelial-to-mesenchymal transition (EMT), reprogrammed metabolism, acidic extracellular pH (pH_e_) and hypoxia can heavily impact treatment outcomes. Therefore, finding new therapeutic targets within the TME of PDAC is an encouraging and potential research direction in order to understand the lack of efficacy of current treatments for pancreatic cancer.

Resistance to cancer chemotherapy or chemoresistance is the innate and/or acquired ability of cancer cells to survive and maintain uncontrolled proliferation leading to tumor progression in spite of the tumor’s exposure to cytotoxic compounds [17]. Chemoresistance also causes disease relapse and metastasis, thus representing a crucial challenge that oncology research has sought to understand and overcome in order to improve the clinical outcome of cancer patients [18,19]. Moreover, tumor heterogeneity promotes a specific tumor response to diverse types of chemotherapy, and the recent literature on pancreatic tumors has confirmed that tumors have a complex microenvironment containing many independent components, each of which exert a unique role in conferring chemoresistance [15,20]. 

Herein, we will describe the main mechanisms of chemoresistance in pancreatic cancer with a main emphasis on the emerging role played by the TME, aiming to provide future directions and to discover novel targets for new therapeutic strategies against PDAC.

## 2. Desmoplastic Reaction in the Pancreatic Tumor Microenvironment

### 2.1. Desmoplasia: The Impact on Tumor Development and Progression

Despite the sizeable improvement in recent years, chemotherapy remains markedly incompetent in improving PDAC patient survival [21]. There are many factors that contribute to the failure of chemotherapy in this pathology, including the occurrence of desmoplasia in the PDAC TME [21].

Desmoplasia, also known as the desmoplastic reaction, consists of a dense ECM together with myofibroblast-like cells, including CAFs and is a fundamental feature of the pancreatic cancer TME [22]. Initially, the role of this phenomenon was overlooked; however, many studies have since demonstrated that during PDAC development, the cancer cells expend a large amount of energy to promote the recruitment, proliferation, and activation of fibroblasts. Consequent to their activation, CAFs are able to deposit ECM components and secrete several types of factors that strongly affect the behavior of cancer cells [23,24,25]. Indeed, pharmacologic inhibition of the desmoplastic reaction in combination with chemotherapy showed better results in inhibiting PDAC progression than chemotherapy alone, thus highlighting desmoplasia as a likely therapeutic target in pancreatic cancer [26,27,28,29]. Desmoplasia can be divided histopathologically into two groups: (i) overproduction of ECM proteins, and (ii) extensive proliferation of the PSCs [30,31]. Hence, the resultant abundant and fibrotic stroma tissue is encompassed by both cellular and non-cellular elements. In this section, we will focus on the non-cellular elements. 

Among all non-cellular components of desmoplasia, the importance of many ECM proteins, namely collagen types I, III and IV, fibronectin, laminin, hyaluronan, as well as the glycoprotein osteonectin [32,33] should be emphasized. Desmoplastic progression derives from the abnormal activation of several intercellular and intracellular signaling processes, such as the transforming growth factor beta (TGFβ), the basic fibroblast growth factor (bFGF), the connective tissue growth factor (CTGF) and interleukin-1β, which by stimulating ECM production drive desmoplastic progression [34,35,36,37,38]. The ECM components can also be divided into two categories: the fibrous proteins, such as collagens, and the polysaccharide chain glycosaminoglycans (GAGs), such as hyaluronan [39,40,41,42]. In the normal pancreas, GAGs structurally function to sustain compressive forces on the tissue, whereas the fibrous proteins act to support the tensile forces on the tissue [43]. On other hand, the marked overproduction of ECM constituents in PDAC has been suggested to be a failed wound healing, leading to fibrosis [43]. The increased deposition of collagen type I, III, and IV in PDAC tissues [44,45,46] is directly linked to the TGFβ/Smad signaling and is a product of the activity of the fibroblasts [47]. Remarkably, in PDAC, the elevated levels of collagen I reduce tissue elasticity and raise interstitial fluid pressure, causing a reduction in drug perfusion [48]. 

**Figure 1:**
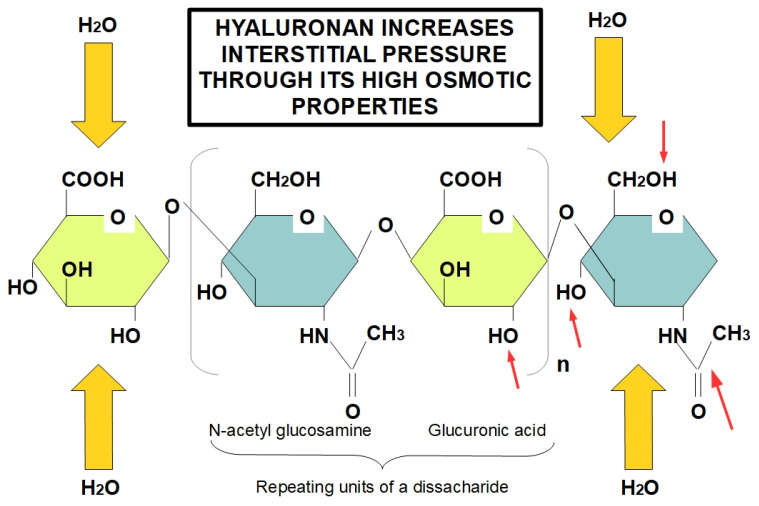
Role of hyaluronan in increasing tissue interstitial pressure. Hyaluronan forms long chains creating a highly osmotic environment that produces edema and increased interstitial pressure. Despite the fact that the diagram only shows a tetrasacharide, hyaluronan is a very lengthy unbranched chain of repeating disaccharides. Red arrows indicate the hydrophilic parts of glucuronic acid and N-acetyl glucosamine, proving the highly hydrophilic ability of hyaluronan. Increased hyaluronan in tumors is an early event occurring in TME, which leads to increased interstitial pressure due to its hygroscopic properties, causing an obstacle to the adequate delivery of chemotherapeutic drugs.

The protein-free GAG, hyaluronan, is also an important component of the ECM, contributing to tissue rigidity and thereby decreasing elasticity [49] and its accumulation within damaged tissue is a product of increased secretion by CAFs in pancreatic cancer [50]. Moreover, hyaluronan maintains its interaction with water molecules, to preserve tissue hydration in normal pancreas [51]. Nevertheless, in the “unhealthy” pancreas the increased deposition of hyaluronan might result in interstitial edema and, consequently, augmented interstitial fluid pressure that, in turn, leads to decreased fluid conveyance [52]. Accordingly, this enhanced interstitial edema together with the absence of an efficient lymphatic system in tumor tissue, results in a remarkable reduction in the exchange of several substances with the bloodstream, including chemotherapeutics [53]. Therefore, this significant deposition of hyaluronan in pancreatic cancer stroma is one of the main features of pancreatic TME responsible for decreased chemotherapeutic penetration (Figure 1) [54,55]. 

Lastly, fibronectin is also a crucial ECM component which binds to adhesion receptors in several cell types [56]. Additionally, it supports cell–ECM interactions and is a key factor for wound healing and development and the maintenance of tissue homeostasis [57,58,59,60,61]. While several cell types including tumor cells and endothelial cells are able to produce fibronectin, fibroblasts are the main producers [59]. The elevated expression of fibronectin is displayed by several solid tumors, particularly in pancreatic cancer [62]. Therefore, the interaction of multiple ECM proteins to produce the desmoplastic reaction in PDAC is a feature which clearly contributes to the pathogenesis and ultimately to chemoresistance. 

### 2.2. The Contribution of Desmoplastic Components towards Chemoresistance

**Figure 2:**
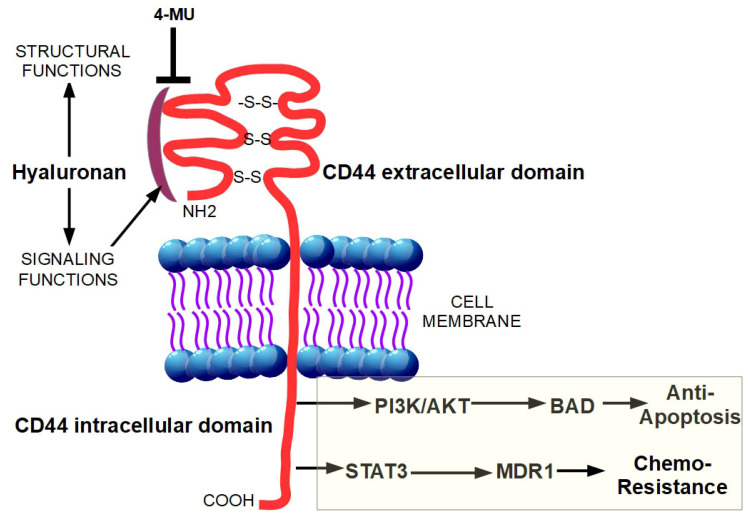
Binding of hyaluronan to CD44 unleashes a pro-tumoral intracellular signaling. The intracellular signaling functions of hyaluronan are triggered after its binding with CD44. This interaction results in the increased expression of the multi-drug resistance protein 1 (MDR1) through STAT3 activation and in the activation of phosphatidylinositol-3-kinase (PI3K/AkT) signaling pathway, causing phosphorylation of Bad, and the subsequent downregulation of apoptosis. The hyaluronan synthesis inhibitor, 4-methylumbelliferone (4-MU), inhibits cell migration, proliferation, and invasion by blocking the interaction between hyaluronan and CD44.

Desmoplasia can promote chemoresistance through several mechanisms which can be divided in two main groups: biological and physiological chemoresistance [31]. Biological chemoresistance can arise from different mechanisms. Target cells can (a) acquire resistance to drug uptake, (b) reduce their sensitivity to drugs by increasing the expression of anti-apoptotic proteins and activating protective mechanisms such as autophagy [63], (c) use DNA repair mechanisms to counteract the drug-dependent destruction of the tumor cell DNA [64,65] and (d) recruit more transporters/proteins responsible for drug efflux, thus preventing their action in the cancer cell. In PDAC, both physiological and biological chemoresistance are present and constitute a considerable problem for effective chemotherapy [31]. Moreover, the non-cellular components of the desmoplastic reaction can contribute to biological chemoresistance. Indeed, the binding of hyaluronan to its receptor, CD44, caused a Stat-3-mediated increase in the expression of the multi-drug resistance protein 1 (MDR1) in pancreatic cancer cell lines [66]. Moreover, the interaction of hyaluronan with CD44 is able to activate the phosphatidylinositol-3-kinase (PI3K/AkT) signaling pathway, which is upregulated in several cancers, resulting in the phosphorylation of Bad, and the consequent downregulation of apoptosis (Figure 2) [67,68].

The ECM components are also involved in physiological chemoresistance and probably to a larger extent than in biological chemoresistance [69]. Physiological chemoresistance is due to poor tissue vascularization in the tumor tissue caused by the overproduction of ECM proteins and the consequent increased interstitial fluid pressure, which together produce a barrier to drug absorption into the target tissue [31]. As mentioned before, collagen type I, III, and IV are highly secreted into the PDAC TME and it was demonstrated, in a PDAC orthotopic xenograft mouse model, that the increased collagen I content decreased the penetration of nanoparticles, resulting in a significant reduction in the response to doxorubicin treatment [70]. Moreover, the collagen-reducing effects of the anti-hypertensive agent, losartan, resulted in a significant augmentation of nanoparticle penetration towards the target cells [70]. However, the decreased collagen I content induced a switch in cancer stem cells (CSCs) from slow growing, avascular-type cells to fast-growing, highly autophagic endothelial-like cells, creating a favorable mechanism for tumor progression and chemoresistance to gemcitabine [63,71]. Altogether, these data suggest that the correct manipulation of ECM collagen composition may be able to enhance the accessibility of drugs to tumor tissues and decrease chemoresistance. However, the role of hyaluronan in physiological chemoresistance is not well established and there is no consensus. While some reports have shown that total tissue hyaluronan content is not correlated with tissue elasticity and hydraulic conductivity [72], another study demonstrated that ECM molecular selectivity is regulated by variation of the hyaluronan content, thus affecting molecule penetration based on charge and size [73]. In line with this second paper, the depletion of hyaluronan in a PC3 xenograft model decreased tumor interstitial fluid pressure and increased vascular area, suggesting that hyaluronan might have an important role in blocking the penetration of chemotherapeutic agents [74]. Regarding PDAC, more studies are needed to disclose whether hyaluronan content may have a role in tissue elasticity and mechanobiology of ECM, and how this can be correlated with drug perfusion and chemoresistance. 

### 2.3. Targeting the Desmoplasia Improves Chemotherapy Outcomes

Several stroma-targeting drugs are currently being tested as new treatment strategies to reduce chemoresistance in PDAC. One of the more appealing targets of the tumor stroma is the potent cytokine TGFβ which regulates developments, differentiation, and homeostasis in mammalian [75]. TGFβ binds to TGFβ receptor 1 or 2 to inhibits cell proliferation, motility, invasion, EMT, and metastasis [76] and its tumor-inhibitory effect is controlled by Smad-dependent TGFβ signaling [77]. Indeed, in human prostate cancer, overexpression of TGFβ1 correlates with collagen I levels, suggesting that TGFβ can be directly linked to the desmoplastic process [35]. Furthermore, knocking down Smad 3 abolishes collagen fibrosis induced by the EMT-regulator Snail, hence corroborating the role of TGFβ as a crucial signaling pathway in the propagation of the desmoplastic reaction [78]. Moreover, Smad4 is frequently mutated in PDAC and this could be one of the reasons for the resistance to the growth inhibitory effects of TGFβ [35]. In this line, some TGFβ receptor 1 inhibitors, such as SB431542 and SB525334, have been developed and tested in combination with gemcitabine, resulting in a higher cytotoxic effect compared to gemcitabine alone due to an increased delivery and penetration of the drugs into the tumor (desmoplastic) tissues [79].

Moreover, the hyaluronan cell surface receptor CD44 has a critical role in pancreatic carcinogenesis [80] such that disruption of the hyaluronan-CD44 complex is a crucial therapeutic target to prevent PDAC drug resistance [80]. Indeed, the compound, 4-methylumbelliferone (4-MU), by inhibiting hyaluronan synthesis and accumulation on cancer cells and their surrounding stroma, blocked cell proliferation, migration, and spreading in several tumor cell types [81,82] and reduced bone metastases in breast cancer (Figure 2) [83]. In both PDAC cell lines and in vivo, 4-MU slowed the development and progression of the disease and also increased tumor response to gemcitabine [84,85,86]. In a similar way, PEGylated human recombinant PH20 hyaluronidase (PEGPH20) acted as a hyaluronan “consumer” enhancing the delivery of chemotherapeutic agents such as doxorubicin and gemcitabine [55,74]. Injection of PEGPH20 into KPC mice tumors rapidly degraded hyaluronan, restored the patency of intra-tumoral vessels, increased vessel diameter and highly reduced the high interstitial fluid pressure within the tumors to normal levels [12]. Importantly, these alterations increased the macromolecules permeability and the combined treatment of gemcitabine with PEGPH20 remarkably improved treatment efficacy by reducing metastases and doubling the median survival time in mice [87].

Further, the enzymatic degradation of ECM components has also been proposed as a possible complementary therapeutic approach against PDAC. Several studies using hyaluronidase showed improved cancer sensitivity to chemotherapeutics and enhanced permeability to drugs in cultured multicellular spheroids [88]. Similarly, collagenases have also displayed beneficial characteristics by increasing the penetration of macromolecules. However, their sensitivity to and stability at physiological pH might be a therapeutic hindrance that has not yet allowed this enzyme to be clinically available [89]. We have proposed a double edged targeting of the hyaluronan-CD44 pathway by combining 4-MU as hyaluronan production inhibitor and bromelain as CD44 inhibitor [90], since this combination has not been experimentally tested yet. Lastly, Hedgehog (Hh) is a signaling pathway that is abnormally activated in most pancreatic cancers leading to cancer initiation, progression and metastatic development [91,92]. More recently, it has been related to the beginning and maintenance of the desmoplastic reaction. Indeed, Hh stimulates the differentiation of myofibroblasts and induces stroma-derived growth promoting molecules [93,94]. There is also some evidence that supports the existence of an interplay between Hh signaling and TGFβ, both being tightly connected with the desmoplastic reaction and involved in fibrosis [94]. Further, it was demonstrated that blocking Hh signaling, both in vitro and in vivo, with the small molecule compound, cyclopamine, markedly improved drug delivery and abrogated pancreatic metastasis [95,96]. Multiple studies have been conducted to verify whether the combined treatment of Hh signaling inhibitors with chemotherapeutic agents can have synergistic anticancer effects [97]. Indeed, the inhibition of the Hh signal with the semisynthetic analogue of cyclopamine, IP-926, reduced the desmoplastic reaction and improved tumor vascularity [98]. A phase Ib trial demonstrated that IPI-926 reduced tumor desmoplasia and increased gemcitabine delivery, such that 31% of patients displayed a partial response and 63% of them showed a reduced expression of the marker Carbohydrate Antigen 19-9 (CA 19-9) in their tumor tissues [99].

In summary, the desmoplastic reaction creates a unique microenvironment, which stimulates tumor growth/progression and forms a “physical barrier” to chemotherapy permeability. Hence, several strategies have arisen to improve chemotherapeutic efficacy by blocking or interfering with the desmoplastic process and enhance tumor penetration, accumulation, and drug distribution. Thus, targeting stroma components of the desmoplastic reaction might be a promising new area of investigation in pancreatic cancer treatment. 

Table 1 lists all the drugs that might be employed against the desmoplastic reaction.

## 3. The Role of Extracellular Acidic pH and Hypoxia in the Resistance to Therapy

In recent years, numerous studies on PDAC have focused on the biologic or metabolic TME, which plays a role in tumor malignancy [155]. The TME represents an important factor that allows tumor growth and survival of the most aggressive cells, leading to chemoresistance and metastatic behavior. The pattern of an acidic extracellular environment together with an alkaline cytosol is considered a hallmark of malignant cancers and is referred to as a “reversed pH gradient” [156]. The intracellular alkalinity confers a proliferative advantage for the malignant cells, while a low nutrient supply, extracellular acidic and hypoxic conditions may contribute to the progression from benign to malignant growth and induce the selection of more aggressive tumor cells capable of withstanding this hostile acidic and hypoxic microenvironment [157]. The new blood vessels (angiogenesis) forming in a tumor are not properly formed and are often twisted and abnormal (convoluted). This defective structure leads to a poor ability to deliver oxygen and remove metabolic waste products, resulting in the development of acidic conditions [158]. In the next subsections, the role of both acidic pH_e_ and hypoxia on PDAC chemoresistance will be discussed.

### 3.1. Extracellular Acidic pH Driving Tumor Progression

The pH_e_ of tumor tissues is often acidic [159] and this phenomenon is the result of multiple factors such as: (i) increased CO_2_ production, which is converted into carbonic acid by membrane Carbonic Anhydrases IX and XII (CAIX and CAXII) [160]; (ii) increased lactate production as a consequence of high glycolytic flux and aerobic glycolysis [161]; (iii) extrusion of this lactate by monocarboxylate transporters (MCTs) [162] and (iv) active translocation of cellular protons into the ECM mainly by sodium-hydrogen exchanger isoform-1 (NHE1) [163]. Hence, all these mechanisms are altered in tumors and lead to low pH_e_ (~6.7–7.1) compared to normal tissues (7.4) [164], and to an alkaline intracellular milieu [164]. This inverted pH gradient may initiate and then drive the further development of the neoplastic process [165]. 

The acidic pH_e_ has a number of important consequences that are directly related to cancer [157,166]. One of the direct consequences of the extracellular acidity is the degradation of the ECM by the activation of proteolytic enzymes, such as metalloproteases and cathepsins [167,168]. This mechanism is associated with migration, invasion, and metastasis [169,170]. In several tumors, including PDAC, the acidic microenvironment promotes the activity of metalloprotease 1, 2, and 9 [171] and drives EMT, invasion, and metastasis [172]. Indeed, the exposition of PDAC cells to low pH_e_ promotes the invasive phenotype [172] by stimulating the expression of mesenchymal markers, such as N-cadherin, and reducing the expression of epithelial markers such as E-cadherin [172]. In this way, acidic pH_e_ may also affect other processes such as angiogenesis, evasion from the immune system, and drug resistance [173,174]. 

### 3.2. Multidrug Resistance and the Acidic Tumor Microenvironment

One of the main therapeutic problems to solve in cancer is the frequent emergence of drug resistance, which can be enhanced by the acidic TME [175,176,177,178].

**Figure 3:**
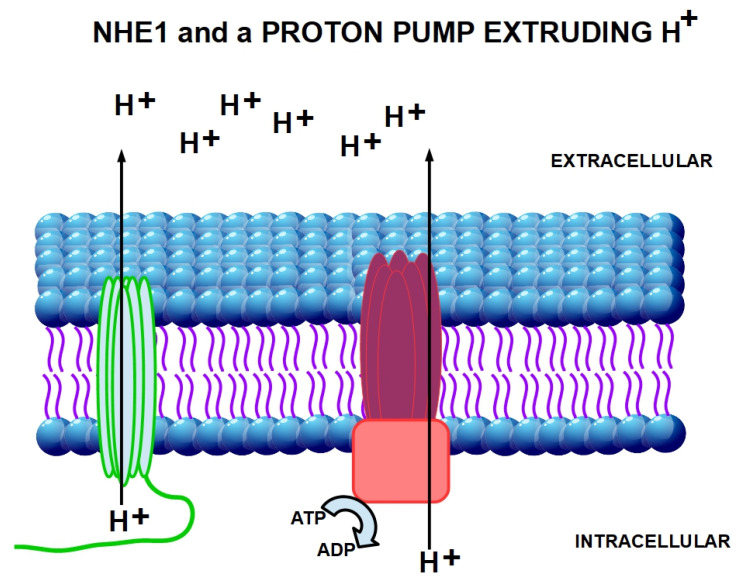
Activity of the proton extruders NHE1 ATPase proton pump. On the left side in green, it is represented the active secretion of cellular protons into the extracellular space by NHE1. On the other side in red, the active extrusion of cellular protons into the extracellular space by V-ATPase proton exporter is shown. Interestingly, both extrude protons against the gradient; however, while proton pumps need energy (ATP) for their activity, NHE1 does not need ATP to achieve the same purpose.

**Figure 4:**
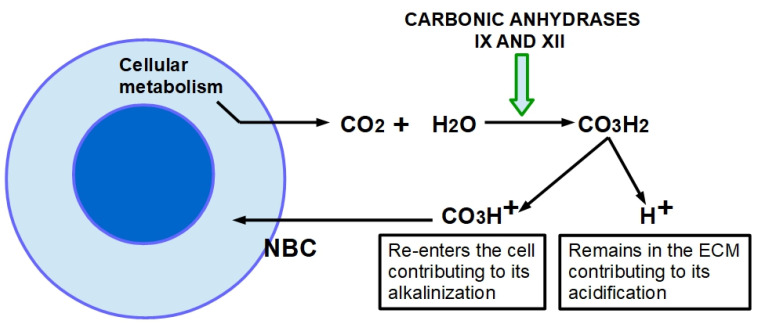
Contribution of carbonic anhydrases to tumor acidification. The cellular metabolism produces an excess of CO_2_ that diffuses from the cell into the extracellular space. Membrane carbonic anhydrases IX and XII convert it in carbonic acid (CO_3_H_2_) through hydration. CO_3_H_2_ spontaneously ionizes into a molecule of ionized hydrogen (proton) that remains in the matrix, contributing to its acidification. The bicarbonate ion is reintroduced into the cell through the activity of the sodium bicarbonate cotransporter (NBC) contributing to cytoplasmic alkalinity.

One of the mechanisms modulating the entry of an ionic drug into the cell is the existence of a reversed extracellular to intracellular pH gradient compared to normal cells. The pH_e_ in normal cells is generally around 7.32 with a slightly more acidic pH_i_, about 7.10–7.20 [179]. This pH_e_ to pH_i_ gradient usually allows weakly basic drugs to enter passively into the cells. This inverted pH gradient has a crucial role in the “ion trapping” hypothesis, which predicts that weakly basic chemotherapeutic drugs such as anthracyclines, anthraquinones and vinca alkaloids will concentrate in more acidic cell compartments, such as the extracellular fluid [180,181]. Therefore, the acid pH_e_ of tumors will effectively hinder weak basic drugs from reaching their intracellular target, thereby reducing cytotoxicity. The ion trapping model also predicts that the acid pH_e_ of tumors will improve the uptake of weak acids such as chlorambucil [182]. The weak basic chemotherapeutic drugs will be seriously impaired by the inverted pH gradient. Acidic and neutral drugs will not be influenced negatively. Multidrug resistant cancer cells display an even higher inverted pH gradient than non-resistant cells [183]. Acidic pH_e_ induces the activity of MDR and a series of related-effects including reduction in cell cycling fraction, selection for an apoptosis-resistant phenotype and increased ion trapping [184,185]. Extracellular acidity was previously considered to be directly related to an excessive lactic acid production, while nowadays it is clear that it is strongly dependent on CO_2_ production and the hyperactivation of proton extruders such as NHE1 and NHE3, vacuolar ATPase proton pump, and membrane enzymes such as CAIX and CAXII and the multidrug transporter P-glycoprotein [186,187,188] (Figure 3 and Figure 4). Therefore, the inhibition of acidity through, for example, sodium bicarbonate or through proton pump inhibitors has been shown to confer greater susceptibility to chemotherapy in tumor cells [189]. 

### 3.3. Hypoxia: A Promoting Factor in Cancer Survival and Proliferation

The term hypoxia means a significant reduction in oxygen tissue levels, which characterizes 50–60% of locally advanced solid tumors, such as PDAC. PDAC tissue has a partial oxygen pressure, with the median pO_2_ of 0–5.3 mmHg (0–0.7%), while the median pO_2_ in the normal pancreas is 24.3–92.7 mmHg (3.2–12.3%) [190,191]. Since the seminal works by Semenza and Wang [192,193], we know that the main molecular mechanism by which oxygen homeostasis is maintained in tissues is regulated by hypoxia-inducible factors (HIFs) [194]. There are three isoforms HIF-1α, HIF-2α, and HIF-3α, and each of them can heterodimerize with HIF-1β and form HIF-1, HIF-2, and HIF-3. HIF-1, the most important, is a heterodimer consisting of a constitutively expressed HIF-1β subunit and an oxygen-regulated HIF-α [195]. During normoxia, HIF-1α protein levels are negatively regulated by the ubiquitin ligase Von Hippel Lindau protein (VHL) which polyubiquitinates HIF-1α for its rapid proteasomal degradation. Hydroxylation of HIF-α by prolyl hydroxylases (PHD1-3) in the presence of oxygen is required to promote its polyubiquitination by pVHL. In contrast, under hypoxic conditions PHDs do not hydroxylate HIF-α which is therefore not ubiquitinated by pVHL. Non-hydroxylated HIF-α accumulates and translocates into the nucleus to dimerize with HIF-β [196]. The heterodimer HIF1α:HIF1β induces a large number of downstream transactivating genes that will allow the cellular adaptation to low oxygen levels. 

**Figure 5:**
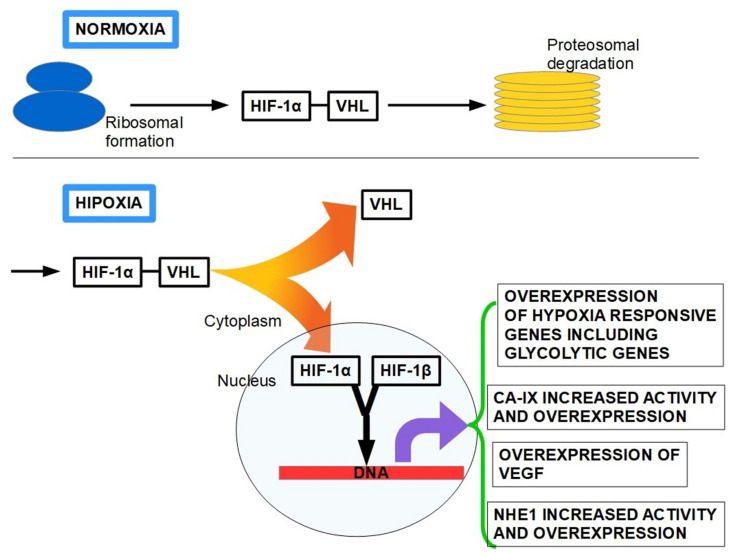
The “fate” of the HIF-1α protein with the oxygen tissue level. **Upper panel**: HIF-1α is unstable in normoxia because due its binding to the VHL protein, it is carried to proteasomal degradation. **Lower panel**: The situation changes under hypoxic conditions. When HIF-1α is released from VHL (stabilization) and translocates to the nucleus, it dimerizes with the constitutional HIF-1β. This dimer acts as a transcription factor for a set of genes that contain a Hypoxia Responsive Element (HRE) sequence in their promoter region. On the right side there are some of the genes that are promoted by the dimer.

Among these genes are those that code for erythropoietin (EPO), enzymes of the glycolytic pathway such as GLUT1, the pro-angiogenic vascular endothelial growth factor (VEGF), the platelet-derived growth factor (PDGF) and NHE1 [197,198,199] (Figure 5). 

The increased expression of VEGF and, therefore, angiogenesis due to HIF-1α could promote the progression of pancreatic tumors [200]. PDAC has been shown to have reduced vascularization compared to the normal pancreatic tissue [201]. Hypoxia in tumors increases with the distance from the nearest capillary blood network, while the proliferative index of tumors decreases [202]. Indeed, tumor growth and size are strongly influenced by vascularization and angiogenesis. The hypoxic environment occurs when there is a high oxygen demand of the cancer cells, and poor lymphatic drainage; consequently, the growth rate of the tumor is greater than the rate of new vessels [203]. Recognized as a hallmark of most solid tumors, hypoxia profoundly influences multiple facets of cancer biology, through the HIF-1 mediated induction of metabolic reprogramming, neovascularization, EMT and metastasis [204,205]. Furthermore, it has been shown that HIF-1α expression increases pancreatic cancer cell motility and metastasis and it is associated with a negative prognosis [206,207]. Importantly, in PDAC cells, hypoxia increases the formation of invasive protrusions, known as invadopodia, and their mediated-focal ECM proteolysis, thereby increasing tumor aggressiveness [208,209,210].

**Figure 6:**
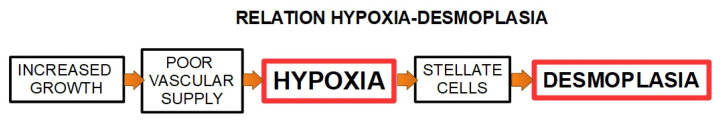
Schematic representation of the relationship between hypoxia and desmoplasia.

EMT is a morphologic cellular program simply defined as the phenotypic transition from a stable epithelial state to a mesenchymal state, with “pro-metastatic” characteristics [211]. Numerous studies have demonstrated that the invasive capacity of PDAC correlates with EMT [212,213] together with their increased ability to migrate while remodeling the ECM barriers (e.g., invasion). These ECM barriers include the epithelial and endothelial basement membranes and the interstitial collagen rich stroma [214]. The hypoxic environment promotes EMT and cells undergoing EMT acquire stem-like features as well as drug resistance in different types of cancer, including PDAC [215]. PDAC stroma is frequently hypoxic, and the PSCs respond to hypoxia by increasing HIF-1α expression [216,217], motility and alpha-smooth muscle actin expression. Furthermore, increased HIF-1α expression stimulates the synthesis of collagen I, fibronectin, and periostin indicating a correlation between hypoxia and the densely fibrotic desmoplastic reaction [201,218] (Figure 6). In addition, the desmoplastic reaction can be considered as an effector of hypoxia, which, in turn, activates invasion and metastasis. Among the various effects of hypoxia on cancer malignancy, there is also the reduction in the effectiveness of chemo and radiotherapy. Since the existence of this malignant interplay between desmoplasia and hypoxia, further studies are required to establish a possible synergetic therapy by using modulators of both pro-carcinogenic events.

### 3.4. Hypoxia: Induction of Chemoresistance in Cancer Cells

Hypoxia is an important hindrance in the development of successful cancer chemotherapies [219,220]. It is believed that hypoxia and HIFs can mediate chemotherapy resistance through mechanisms such as the extrinsic resistance, the regulation of drug efflux, metabolic reprogramming, alterations in apoptosis and cell survival, and induction of stemness. Recent reports have demonstrated that hypoxia selects for cells with increased apoptotic resistance to chemotherapeutic drugs by the overexpression of Bcl-2 and/or the downregulation of proapoptotic proteins (BNIP3, NOXA, and NIX), together with a diminished drug sensitivity through upregulation of the MDR protein expression [221,222,223,224]. Indeed, in PDAC cells it was observed that hypoxia enhanced apoptosis resistance induced by gemcitabine via PI3K/Akt/NF-kappa B pathways and partially through the MAPK(Erk) signaling pathway [225]. Other studies have shown an association between HIF expression and the regulation of drug efflux. HIF-1α stimulates the expression of the MDR1 gene coding for P-glycoprotein 1, a predominant membrane transporter associated with chemotherapy resistance [226] which is also involved in the reduction of intracellular level of drugs, such as paclitaxel and anthracyclines [227].

Another mechanism by which hypoxia induces chemoresistance is through metabolic reprogramming and the modulation of reactive oxygen species (ROS) production [228]. For example, Mayer Y. Abdalla et al. suggested that in pancreatic cancer cells hypoxia could upregulate Heme oxygenase 1 expression [229]. Inhibiting Heme oxygenase 1 with zinc protoporfiphyrin and tin protoporphyrin IX increased both ROS production and apoptosis, thus sensitizing pancreatic cancer cells to gemcitabine [229]. Moreover, the glycolytic enzyme enolase 1 alters ROS production and promotes chemoresistance [230]. 

### 3.5. Therapeutic Strategies Targeting the Acidic Extracellular pH and Hypoxia

As the contribution of the TME to the lethal outcomes of PDAC is substantial, the complex relationship between acidic pH_e_, hypoxia, ROS, and treatment resistance requires further research. Consequently, hypoxia and HIF-1α are key factors to have in mind while studying new therapeutic strategies. One of the possible strategies might be improving tumor perfusion through anti-fibrotic therapies, since PDAC is one of the most desmoplastic epithelial tumors [231]. Indeed, the addition of PEGPH20 to nabpaclitaxel/gemcitabine doubled the progression-free survival in a group of patients who had tumors with high hyaluronic-acid content [232]. However, it is necessary to better understand the functional characteristics of the stroma in order to be able to apply therapy to different cell types such as PSCs, that are the dominant producers of VEGF and the main contributors to the fibrotic/hypoxic milieu through abnormal ECM deposition [201]. Another strategy to target hypoxia-induced pathways in PDAC is by inhibiting HIF-1α signaling. A plant-derived agent, triptolide, has been shown to decrease both HIF-1α levels as well as the CSCs subset of PDAC [233,234]. One of its derivatives is now being tested in advanced gastrointestinal cancers including PDAC [235]. While the well-known cardiac glycoside, digoxin, which effectively inhibits HIF-1α synthesis at a relatively low concentrations, could be used as a sensitizer to reverse chemoresistance in PDAC, there are no registered clinical trials testing this hypothesis [236]. In a recent study, Lang and colleagues showed that a compound extracted from melphalan (PX-478) combined with arsenic trioxide could be a promising strategy to promote ROS-induced apoptosis in the treatment of PDAC [237]. Another therapeutic mechanism could be to block the signal transduction that upregulate HIF-1α expression [238]. In this regard, both Mek/Erk pathway and mTOR play a role in the regulation of HIF-1 expression. While the agent, everolimus, alone had minimal clinical activity in gemcitabine-refractory PDAC [239], when in combination with capecitabine, displayed a moderate clinical response. This indicates that the combination of mTOR inhibition with another targeted therapy or cytotoxic agent may show clinical benefits [240]. Moreover, the high production of ROS stabilizes HIF-1α and, consequently, the attenuation of this production with antioxidants or superoxide dismutase can inhibit tumor growth and metastasis [241,242]. However, decreasing ROS levels also reduces the effects of chemotherapeutic drugs [243]. In order to improve or develop new therapies in PDAC, it could be important to identify a subset of patients and cancer cell subpopulations expressing and hypoxia markers for HIF-1α -interfering substances. A better understanding of the mechanisms related to acid microenvironment and hypoxia is the basis for clinical and therapeutic improvements of PDAC in the future.

## 4. The Tumor Metabolic Microenvironment Promotes Resistance against Chemotherapy

### 4.1. Metabolic Rewiring and Nutrient Scavenging in Cancer Cells

Lack of vascularization in the tumor core not only causes hypoxia, but it also triggers metabolic stress due to nutrient deprivation. In the last two decades, metabolic reprogramming has been recognized as one of the hallmarks of cancer cells [244]. It has been proposed that cancer cells display a higher metabolic rate than their normal counterparts. Moreover, tumor cells are able to use glucose, glutamine, fatty acids, and even other amino acids as substrates to support their energy needs [245,246]. Cancer cells have the ability to reprogram their metabolism through several different ways with the purpose of supporting their unrestricted proliferation. As a result of their metabolic reprogramming, cancer cells quickly adapt to the characteristic physical changes occurring in the microenvironment, which reciprocally contributes to the heterogeneous cellular metabolic landscape of the tumor niche [247]. The most-well known metabolic alteration in cancer cells is the Warburg effect which postulates that cancer cells have enhanced aerobic glycolysis and less glucose oxidation compared to their normal counterparts [248]. Therefore, tumor cells have a higher flux through the pentose phosphate pathway, the anabolic side branches of glycolysis [249]. Indeed, the most important metabolic change observed in cancer cells is the shift of fuel use through anabolic pathways, with the objective to provide the cells with enough substrates to increase biomass [250].

As mentioned before, it is well known that PDAC is a hypovascular tumor, which results in inadequate tumor perfusion, meaning less availability of glucose, amino acids, and lipids [251]. To overcome this obstacle, cancer cells frequently exploit various scavenging strategies to harvest macromolecules from the microenvironment and break them down in the lysosome, creating substrates for ATP generation and anabolism [252]. Autophagy is the major mechanism used for nutrient scavenging by cancer cells. Moreover, it is a cellular mechanism which culminates in the lysosomal degradation of intracellular material and provides metabolic and cellular homeostasis through the recycling of cytoplasmic elements to cellular building blocks [253]. The subsequent autophagosome after merging with the lysosome delivers the recycled material back to the cytosol, via the degradation of its cargo [254,255]. Indeed, autophagy is a key cellular process since its dysfunction is associated with several disorders, including neurodegenerative diseases, inflammation, and cancer [256]. In PDAC, it has been demonstrated that autophagy is increased compared with normal cells/tissues [257]. Metabolism has been proposed as the possible mechanism with which autophagy may interact and the result of this interplay contributes to PDAC progression. This interplay relies on the ability of the autophagy to feed the metabolism by supplying recycled intracellular components, and this promotes a pro-tumorigenic effect [257]. Since autophagy is able to degrade a substantial range of substrates, it is evident that this cellular mechanism has the potential to fuel almost all pathways in central carbon metabolism. Therefore, the resultant metabolic plasticity confers to these tumors a survival advantage in the harsh TME which is characteristic of PDAC [258]. In PDAC cells, autophagy inhibition results in an impaired mitochondrial function, causing a reduced oxidative phosphorylation and a consequent fall in ATP levels [258]. 

Importantly, autophagy is not able to form a new biomass, since these cells are also degrading themselves [253]. To overcome this disadvantage, PDAC cells also rely on other lysosomal-dependent pathways to fuel their high metabolic requirements. Macropinocytosis consists in the engulfment and uptake of large amounts of extracellular fluid, containing protein, lipid, virus and bacteria [259]. This mechanism culminates with the release of the digested cargo into the cytosol; however, contrary to autophagy, the breakdown of this macromolecular cargo into their monomeric constituents will create a new intracellular source of diverse nutrients, leading to an increased biomass [260]. Interestingly, nutrients obtained by macropinocytosis have been demonstrated to display a crucial role in PDAC metabolism. Some reports demonstrated evidence of macropinocytosis occurring in human PDAC tumors [261]. It was shown that PDAC cells can take up and degrade collagen from the ECM through macropinocytosis [262] and, importantly, the collagen-derived proline contributed to central carbon metabolism and promoted PDAC cell survival even under nutrient poor conditions [262].

Overall, these studies demonstrate the critical role of recycling and scavenging mechanisms in modulating PDAC metabolism and its subsequent uncontrolled growth in the harsh tumor microenvironment. 

### 4.2. Fuel Source Plasticity towards Resistance to Therapy

#### 4.2.1. Glucose

Glucose is the primary metabolic energy source for sustaining several biochemical processes, including cancer cell proliferation by both supplying carbon for anabolic reactions and by generating ATP [263]. Cancer cells essentially rely on the “Warburg effect”, in which cells depend on mitochondrial oxidative phosphorylation to produce energy for cellular processes, rather than on mitochondrial aerobic glycolysis [264]. Although this change in glucose metabolism is less efficient in producing cellular energy, it confers a survival advantage to cancer cells via a rapid increase in ATP production [265]. Recently, a new two-compartment model has emerged, the “reverse Warburg effect”, in which cancer cells stimulate aerobic glycolysis in the stromal cells, whose glycolysis end-products are then used by cancer cells to feed mitochondrial oxidative phosphorylation [266]. The heterogeneity of PDAC TME might also be partially explained by this model, where the glycolytic differentiated cancer cells could provide substrates to oxidative CSCs thus creating a symbiotic relationship with them [267].

The shift towards enhanced glycolysis as a supplier of ATP, reduces neovascularization creating an adverse milieu, where both oxygen and nutrients are restricted [190,268]. These extreme microenvironmental conditions exert a drastic selective pressure on cancer cell growth and survival leading to the expansion of the most aggressive cellular clones. However, to overcome these stressful conditions, PDAC cells are forced to reprogram their metabolism with the objective to handle their bioenergetic demands for their proliferation and spread towards less harsh environments [217]. These metabolic changes are directly linked to the aberrant activity of specific oncogenes which drive the switching of nutrient preference [269]. Mutations in *KRAS* and other oncogenes (such as *MYC*) and tumor suppressors (*TP53*, *RB* and *PTEN*) have been identified as the principal drivers of PDAC reprogramming cellular metabolism towards enhanced cancer growth [270]. 

Indeed, oncogenic *KRAS* supports substantial alterations in the glycolytic pathway, including the upregulation of the glucose transporter (GLUT1) as well as the enzymes hexokinase (HK1 and HK2), phosphofructokinase-1 (PFK1) and lactate dehydrogenase (LDHA), aiming to satisfy the increased necessity for glucose required in PDAC [271,272]. Moreover, another metabolic advantage promoted by KRAS is the synthesis of monomeric constituents essential for cancer cell proliferation, namely amino acids, and nucleic acids, by deviating glucose toward anabolic pathways, including the pentose phosphate pathway [271]. As expected, a significant reduction in glucose uptake and consequently in the glycolytic flux was observed after silencing the oncogenic *KRAS* gene in tumors [271,273]. 

Another metabolism-related gene and one of the major genetic alterations in pancreatic cancer is *TP53*, which is mutated in >70% of all PDAC cases. TP53 contributes to the glycolytic shift via upregulation of GLUTs, particularly GLUT1 [274]. At the end of the glycolytic pathway, the generated pyruvate is mainly converted to lactate instead of undergoing oxidative phosphorylation, a change driven together with *KRAS*-mediated upregulation of LDHA [271]. Moreover, LDHA plays a key role in the renewal of the glycolytic cofactor NAD+, supporting the increased NAD+/NADH ratio and allowing an intensified glycolysis in cancer cells [275]. These common observed metabolic changes in cancer cells provide the necessary substrates to maintain the enhanced glycolytic flux and lactate production. Interestingly, lactate was recently discovered to be more than a waste product of glycolytic metabolism as it can be used as an energy source [276]. According to this mechanism known as “lactate shuttle”, glycolytic cancer cells generate lactate, which is extruded to the extracellular environment by the lactate transporter MCT4. Lactate is then taken up by oxidative cancer cells expressing MCT1, thus conserving the available glucose for glycolytic cancer cells [277,278]. Last but not least, microenvironmental acidosis due to protons extruded by the cell via these lactate/H^+^ cotransporters, contributes to the suppression of immune cells by supporting chronic inflammation, while suppressing the T-cell mediated adaptive immune response [174]. It is now becoming clear that the co-presence of lactate and acidic pH, being closely linked with chemoresistance, is associated with poor prognosis, metastasis and more aggressive tumor phenotypes [279]. 

#### 4.2.2. Glutamine and Other Amino Acids

Another challenge faced by PDAC cells is the lack of amino acids in their characteristic harsh nutrient-poor microenvironment. To counteract this amino acid depletion and support their metabolic demands, cancer cells utilize different processes. The most abundant free amino acid in humans is glutamine and recent studies have demonstrated that glutamine can be used by cancer cells to support anabolic processes to fuel proliferation [280]. Indeed, the TCA cycle is continuously supported by glutamine-derived carbon in cancer cells [281]. Recently, it has been demonstrated that PDAC cells are able to use a non-canonical pathway of glutamine to satisfy their needs for tumor growth [282]. Indeed, most KRAS-mutated PDAC cells utilize glutamate dehydrogenase to convert glutamine-derived glutamate into α-ketoglutarate in the mitochondria to fuel the TCA cycle. On the other hand, in these cells mitochondrial glutamine-derived aspartate is transported via the mitochondrial uncoupling protein 2 (UCP2) from the matrix to the cytosol, where it is converted into oxaloacetate by aspartase transaminase [283]. One of the resultant products of this pathway is NADH, which helps in the maintenance of the cellular redox state, as well as in the production of metabolites needed for a de novo synthesis of macromolecule and lipids [284]. Interestingly, knocking down UCP2 in KRAS-mutated PDAC cells, results in strong suppression of tumor growth both in vitro and in vivo [273]. Furthermore, a strong relationship between glucose and glutamine metabolism has been reported, in which cell survival in the acidic TME triggered by lactate production from heightened glycolysis relies on amplified expression of aspartate transaminase and on the above mentioned non-canonical glutamine pathway [285]. Indeed, disruption at multiple steps of this pathway results in redox imbalance and diminished cellular proliferation [284]. Besides glutamine, several reports have shown a strong correlation between elevated plasma amino acids (leucine, isoleucine and valine) and pancreatic cancer risk [286]. Importantly, an intensified consumption of amino acids may arise about 10 days before PDAC diagnosis, suggesting that this increased plasma amino acids concentration should be considered as pre-diagnostic and diagnostic tool [287]. All these findings suggest that the level of some amino acids could be used as a tool to stratify PDAC patients after diagnosis, also considering the significant differences in their expression observed between normal versus malignant tissue and different disease stages [287].

The bidirectional interaction cancer cells–stromal cells in PDAC stroma represents another source of amino acid uptake for PDAC cells. Indeed, a large portion of the alanine, used by cancer cells to fuel their glutamine and glucose metabolism is secreted by PSCs [288,289]. Moreover, CAFs display an up-regulated amino acid catabolism and are able to fuel PDAC cells with branched-chain α-ketoacid [290]. This cellular communication between PDAC cells and the surroundings is extremely dependent on the expression of the L-type Amino Acid Transporter or the Cystine/Glutamate Exchanger, linking the expression of amino acid transporters to poor prognosis and drug resistance [291,292].

Recently ferroptosis, a regulated cell death mediated by iron accumulation and lipid peroxidation, has been recognized to have an important role in PDAC progression and treatment response; however, the underlying mechanisms are still not completely understood [293,294,295,296,297,298]. Interestingly, cystine handling/metabolism and particularly the cystine transporter (xCT) has recently been recognized to play a role in PDAC ferroptosis [299,300]. xCT knockdown [292] or inhibitors [301,302] have been studied as therapeutic compounds although there is some evidence that it could be a double-edged sword [303,304]. This is an interesting aspect of overcoming therapeutic resistance that deserves further study. 

#### 4.2.3. Lipids and Fatty Acids

Lipids and fatty acid metabolism are becoming a promising object of research in cancer metabolism. Indeed, lipid synthesis is involved in sustaining the bilayer lipid membrane formation, especially controlling the fluidity and shape of the membrane, producing a denser membrane which might diminish the uptake of anticancer drugs and promote chemoresistance [305]. Moreover, lipids are involved in signal transduction by building lipid rafts regulating protein recruitments and interactions, as well as by the production of lipidic signaling molecules [305]. Lastly, ATP production through beta-oxidation is necessary to support cancer cell division and proliferation and it is highly dependent on lipidic metabolism [305,306,307,308]. Fatty acids have a vital role in supporting PDAC development and cancer cell growth [309]. Compared to the normal pancreas, the pancreatic TME is thought to be deficient in lipid levels, which can be explained either by the scarcity of lipids or excess utilization [310]. Curiously, it has been observed that PDAC cells have an augmented tendency to take up exogenous cholesterol through elevated levels of LDLR [311]. Additionally, there is also evidence in both in vitro and in mouse models of PDAC that a high fat diet is able to increase PDAC cell growth [312,313]. Overall, these reports support the theory that exogenous lipids are beneficial for PDAC tumors.

In PDAC, normal human pancreatic ductal epithelial cells with the mutated *KRAS* oncogene enhance their lipid uptake from the environment [314]. Furthermore, fatty acid synthase (FASN), a pivotal enzyme in fatty acid synthesis, is upregulated by EGFR/ERK signaling and its inhibition is detrimental to PDAC cells [315]. It seems that the enhanced ability of PDAC cells to either take up or synthetize lipids and fatty acids plays a key role in cancer progression and development. In fact, high transcriptional levels of FASN or the low-density lipoprotein receptor (LDLR), responsible for the uptake of cholesterol, are correlated with poor overall survival or reoccurrence in PDAC, respectively [316]. 

It has been shown that lipid synthesis accounts for 75–90% of the cellular palmitate pools in PDAC [314]. Most cancer types need high levels of acetyl-CoA to sustain their high rates of fatty acid synthesis and to maintain this high demand there are different sources of acetyl-CoA production [317]. The first process of acetyl-CoA synthesis is due to the citrate metabolism. Citrate is exported from mitochondria via the tricarboxylate transporter SLC25A1 and in the cytosol is metabolized by ATP citrate lyase to produce acetyl-CoA and oxaloacetate [318]. Another pathway for acetyl-CoA production is related to acetate metabolism obtained from the diet or other external sources. Alternatively, MCT1/4, which are mainly recognized as transporters of lactate and pyruvate, were discovered to act also as transporters of acetate into the cell [319,320]. Indeed, these transporters are upregulated in PDAC, and it is believed that, besides being associated with lactate shuttle these transporters might also be associated with de novo lipid synthesis [321].

Additionally, it has been demonstrated that PDAC cells consume acetate from the environment and the ability to take it up is determined by the expression of acyl-CoA synthetase short-chain 2 [322]. The following steps in fatty acid synthesis consist of the action of the enzyme acetyl-CoA carboxylase (ACC), converting acetyl-CoA into malonyl-CoA, producing C16 palmitate through reactions catalyzed by FASN. The resultant palmitate has multiple intracellular functions, involving the regulation of signaling networks in PDAC. Therefore, interfering with these networks might help to mitigate PDAC growth [323]. 

Besides the above-mentioned direct effects, there is an increased complexity of lipid metabolism in PDAC, since some lipids and fatty acids have a role in supporting PDAC tumor progression while others seem to have anti-tumor effects. Indeed, while obesity and dietary factors are established risk factors for PDAC, there are some fatty acids that displayed anti-tumor effects by decreasing tumor cell proliferation [324]. Furthermore, polyunsaturated fatty acids of the n-6 variety are considered to promote the proliferation of PDAC cells, whereas n-3 polyunsaturated fatty acids have the opposite effect [325]. Overall, it is mandatory to further understand the mechanisms involved in the regulation and response to different fatty acids with the objective to disclose and identify novel therapeutic and treatment approaches for PDAC treatment.

**Figure 7:**
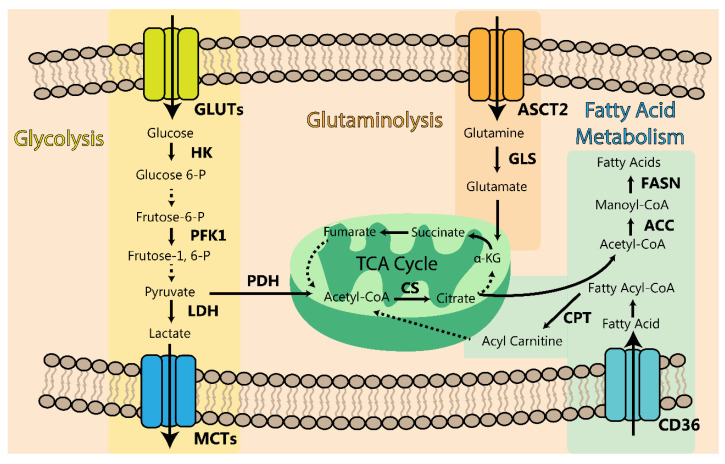
Schematic representation of the discussed metabolic pathways in PDAC. The glycolytic pathway (yellow shade), glutaminolysis (orange shade), and the fatty acid metabolism (blue shade) are represented. The enzymes and transporters (bold) are the key intermediated targets which can be envisaged for new promising therapeutic strategies. Dashed arrows indicate more reactions not explored in the review. Legend: glucose transporters (GLUT1 and GLUT3), hexokinase (HK), phosphofructokinase 1 (PFK1), pyruvate dehydrogenase (PHD), lactate dehydrogenase (LDH), monocarboxylate transporters (MCT4 and MCT1), fatty acid transporter CD36, fatty-acid synthase (FASN), acetyl-CoA carboxylase (ACC), carnitine palmitoyl transferase (CPT), citrate synthetase (CS), ASCT2 (glutamine transporter) and glutaminase (GLS).

Figure 7 summarizes all the metabolic pathways discussed above.

### 4.3. Targeting Metabolism to Overwhelm Chemoresistance

As described above, multiple metabolic changes, driven by genetic and epigenetic factors, have been associated to drug effects and clinical outcome, strengthening the hypothesis that cancer metabolism is deeply related with chemoresistance [326]. Moreover, metabolic reprogramming can promote several key tumor features, thus shaping cancer cell differentiation, proliferation and/or apoptosis, as well as therapeutic response [327]. Various enzymes and transporters that participate in the multiple metabolic pathways have been implicated in stimulating the drug resistant phenotype. The next sub-sections will discuss, in detail, the most important alterations leading to chemoresistance and the possible benefits of manipulating the metabolism as a complementary therapy in PDAC.

#### 4.3.1. Glucose Transporters (GLUT Family)

GLUT1 is an ATP-independent transmembrane protein, and it is responsible for the facilitated diffusion of glucose across the plasma membranes of mammalian due to a glucose gradient from the extracellular compartment to the cytoplasmic compartment [328]. Its expression is higher in PDAC compared to normal pancreas and it is strongly correlated with PDAC stages, since the levels of GLUT1 increase as the disease progresses and becomes more aggressive. Indeed, high GLUT1 expression in resected tumors correlates highly with tumor size, nodal involvement, and shorter patient survival, suggesting that GLUT1 may serve as a prognostic marker [329,330,331,332]. Some reports have demonstrated the ability of apigenin, a dietary flavonoid, to inhibit the GLUT1 action as a contributor to cancer progression [333]. In PDAC, several in vitro studies support the beneficial effect of using the inhibitor of GLUT1 activity, apigenin, to reduce proliferation and angiogenesis by interfering with the PI3K/Akt and VEGF-HIF-1α pathways, respectively [334,335,336]. More studies are needed specially using animal models to confirm the tumor suppressor activity of apigenin and its possible potential as a future complementary chemopreventive agent for pancreatic cancer.

#### 4.3.2. Hexokinase (HK)

HK is the enzyme responsible for the first glycolytic step and has two isoforms, with HK1 predominantly found in cytoplasm, whereas HK2 is mainly present in mitochondria [337]. HK2 is upregulated in multiple cancer types and has the ability to prevent mitochondrial apoptosis through its direct insertion in the mitochondrial outer membrane [245]. Moreover, the expression of HK2 is elevated in PDAC metastatic cells, demonstrating its relationship with the aggressiveness and progression of the disease [338]. Cancer associated-survival pathways such as PI3K/Akt/mTOR pathway are able to stimulate HK2 in cancer cells, inducing drug resistance [339]. HK2 is considered to be a crucial target for anticancer drug therapy due to its role in modulating apoptosis and cellular bioenergetics. The non-specific HK2 inhibitor, 3-bromopyruvate, can reduce ATP reserves and, therefore, reverse chemoresistance in pancreatic cancer cells [340]. It should be noted that elevated ATP levels resulting from increased glycolysis are also linked to activation of HIF-1α and chemoresistance [341]. Regarding the CSCs, which have the most aggressive and drug resistant phenotype, the utilization of 3-bromopyruvate to inhibit the glucose turnover sensitized this aggressive population to gemcitabine [342]. Moreover, this inhibitor has also been tested in vivo in an orthotopic mouse model of human pancreatic cancer, where it was found that ultrasound-guided delivery of 3 bromo-pyruvate blocked tumor progression through a decrease in proliferative potential and apoptosis induction [343]. 

#### 4.3.3. Fructose Biphosphate Aldolase

Another enzyme overexpressed in PDAC is the fructose biphosphate aldolase (FBA), which encompasses the step of converting fructose 1,6-biphosphate into glyceraldehyde-3-phosphate (G3P) and dihydroxyacetone phosphate [344]. Both the overexpression of FBA and high levels of G3P are known to downregulate apoptosis by suppressing caspase-3 activity [345]. Despite the strong evidence that both proteins may have a role in PDAC progression and chemoresistance, there are no studies exploring this area. However, in other tumor types it was found that FBA correlates with the aggressiveness of the tumor [346]. Therefore, the use of either FBA or G3P as targets for new chemicals should be encouraged, with the objective to reduce the cancer-associated drug resistance. 

#### 4.3.4. Lactate Dehydrogenase (LDH)

Another important enzyme is LDH since it is responsible for the conversion of pyruvate into lactate, the endpoint of fermentative glycolysis. LDH is overexpressed in pancreatic cancer and promotes the growth of pancreatic cancer cells [161]. Moreover, the expression of this enzyme is closely associated with aggressiveness and poor prognosis, suggesting that it might be a good prognostic marker [347]. Lactate dehydrogenase 5 (LDH-5) catalyzes the reduction of pyruvate by NADH to form lactate, hence maintaining a constant availability of NAD^+^ to support glycolysis [348]. Tumor-dependent upregulation of the LDH-5 level was observed in pancreatic cancers and was found to correlate with metastases, tumor stage, recurrence of the tumor, and patient survival [349]. On the other hand, a recent report on new LDH inhibitors in PDAC demonstrated a synergistic effect of LDH inhibition with gemcitabine, probably by increasing the expression of deoxycytidine kinase (dCK), which is an important mediator of apoptosis-induced gemcitabine [350]. Moreover, the inhibition of LDH both in vitro and in vivo, with the small-molecule inhibitor, FX11, induced oxidative stress, cell death and inhibited tumor progression [351,352]. Considering these promising results, a deeper analysis is needed to disclose the clinical potential of the inhibition of LDH as a new encouraging target for PDAC treatment. 

#### 4.3.5. Pyruvate Kinase (PK) and Monocarboxylate Transporters (MCTs)

PK is a tetrameric enzyme divided into four subtypes (M1, M2, L and R), which are diversely expressed in various cell types [353]. PKM2, the isoform that converts phosphoenolpyruvate (PEP) and ADP into pyruvate and ATP, is the rate-limiting enzyme of glycolysis [354]. This enzyme is overexpressed in several types of cancer, possibly to drive enhanced glycolytic fluxes due to its high affinity for PEP. In pancreatic cancer cells, PKM2 promotes cell survival and invasion, especially under metabolic stress by enhancing Warburg effect and modulation of ROS production [355,356], thus becoming directly associated with pancreatic chemoresistance [357]. By increasing aerobic glycolysis, PKM2 maintains increased levels of lactate, which is known to have an important role in driving cancer metastasis [358]. Indeed, the knockdown of PKM2 significantly sensitizes pancreatic cancer cells to gemcitabine, by activating several caspase family members leading to apoptosis [359]. Lactate plays also a role as a signaling intermediate in hypoxic conditions leading to the induction of survival pathways [360]. Furthermore, lactate is also able to attenuate the immune response and, in particular, tumor-derived lactate can prevent the response of human T-cells, which are the predominant infiltrated immune cell in the human PDAC TME [361]. In addition, the presence of MCT4 in high levels defines a glycolytic subtype of pancreatic cancer which is correlated with a poor prognosis and outcome [362]. Therefore, the inhibition of the lactate transporters of the MCT family is being considered as a promising potential therapeutic option for cancer treatment, including PDAC [363,364]. 

#### 4.3.6. Fatty Acid Transporter CD36

The intracellular uptake of fatty acids is highly dependent on the presence and activity of the transporter CD36 [365]. In PDAC, CD36 is able to influence gemcitabine resistance through the regulation of anti-apoptotic proteins [366]. Indeed, it has been suggested that PDAC patients with elevated CD36 expression have lower overall survival and recurrence-free survival rates compared to patients with reduced expression [366]. Overall, CD36 levels can be suggested as a prognostic factor for more aggressive stages of PDAC and the use of anti-CD36 strategies together with chemotherapy seems to be an encouraging therapeutic strategy and studies on this topic should be strongly encouraged.

#### 4.3.7. Fatty Acid Synthase (FASN) and Carnitine Palmitoyl Transferase 1A (CPT1A)

PDAC cells are characterized by an increased expression of some lipid metabolism enzymes and transporters, such as FASN and CPT1A, respectively. Firstly, FASN was discovered to be upregulated in high-grade pancreatic tumors, suggesting that this enzyme might be helpful as a prognostic factor and a promising therapeutic target [367,368]. Indeed, the levels of FASN are correlated with both chemotherapy resistance, especially to gemcitabine, and to radiation resistance in PDAC [357,369]. In line with this, the use of FASN-siRNA or a FASN inhibitor, such as orlistat, decreased gemcitabine-associated resistance in PDAC cells [370]. Although these findings demonstrated a relationship between FASN and chemotherapy resistance, the underlying mechanisms are not yet elucidated, and further studies are needed. Lastly, the inhibition of the enzyme responsible for the entrance of fatty acids via mitochondria (CPT1A), by using etomoxir has been shown to restore the sensitivity of pancreatic CSCs to gemcitabine [371]. Altogether these data indicate that inhibition of FASN and CPT1A might represent important therapeutic targets. Further studies are essential to better understand the role of both enzymes on cancer metabolism reprogramming and whether this plasticity is important for the cancer cell to supply ATP under energy stress. 

#### 4.3.8. Clinical Trials: First Evidence in PDAC Patients

Some metabolic inhibitors of glycolysis have reached clinical trials for PDAC treatment. 2-deoxyglucose (2-DG), 3-bromopyruvate and lonidamine are HK inhibitors that were tested in early phase clinical trials, including some trials in PDAC patients [370]. Moreover, an LDH inhibitor, FLX11, was shown to reduce growth of both lymphoma and PDAC xenografts [372]. Despite the fact that PDAC has multiple metabolic modifications to meet the needs of unrestrained proliferation, there are still several aspects to be explored, such as how the complexity of the TME shapes metabolism and might contribute to carcinogenesis and progression of the disease. PDAC is characterized as a highly hypoxic tumor with significantly impaired drug perfusion and low nutrient availability [373,374]. Aberrant metabolism allows cancer cells to acquire resistance to standard treatments by modulating apoptosis, angiogenesis, invasion, and by affecting drug transport and targets. Several studies have demonstrated encouraging results by combining chemotherapy with metabolic inhibitors, where a synergistic effect for PDAC treatment was found [142,143,309,311,375,376]. However, most inhibitors are still in the preclinical phase and the glycolytic pathways are the only ones that have been exhaustively studied and are, at the moment, the most promising for targeting as alternative PDAC treatments. Further studies are necessary especially disclosing the possible role of other metabolic pathways such as glutaminolysis and other amino acids and fatty acids and lipids pathways, on the development and progression of PDAC with the objective to discover novel targets for PDAC treatment. 

## 5. Conclusions

Despite the great effort made to date in the scientific research, PDAC still poses a major therapeutic challenge. So far, only small improvements have been achieved in the diagnosis and management of patients with this disease. PDAC TME stroma is characterized by an intense desmoplastic reaction, hypoxia, acidosis, high interstitial pressure and heterogeneity. The desmoplastic reaction is strongly related with cancer invasion, progression, and metastasis through a complex crosstalk between malignant and stellate cells. Moreover, desmoplasia combined with high interstitial pressure obliterates the poorly vascularized tumor increasing further hypoxia, which is usually found in all tumors. Altogether, these events create a mechanical barrier for the access of chemotherapeutic drugs. 

In the last decade, much progress has been made and it has been found that extracellular acidosis supplies a beneficial environment for cancer cells, by creating a chemical barrier to immune surveillance and chemotherapy. This acidic environment results mainly from the dysregulated metabolism of cancers. The secreted metabolic end-products and cytokines stimulates the interaction between cancer cells and stromal cells, which contribute to the tumor progression and aggressiveness. Indeed, the acidic microenvironment functions as a trigger to the metastatic cascade, stimulating EMT, ECM degradation, and tumor cell migration. 

Due to the complex TME which comprises the mechanisms discussed in this review, namely, desmoplastic reaction, hypoxia, lack of vascular access, low extracellular pH, and the production of drug extruder proteins, working together or separately, PDAC still displays low chemosensitivity to most of the chemotherapeutic drugs. The complexity of the components of TME makes evident their importance in immune system suppression and tumor progression. Indeed, since PDAC drug resistance relies on this specific hostile microenvironment and metabolic reprogramming, this offers potentially innovative strategies for treating patients with PDAC in the future. 

Therefore, targeting desmoplasia and/or cancer metabolism in combination with other targeted agents or cytotoxic compounds represent promising therapeutic strategies that could be beneficial for PDAC.

## Figures and Tables

**Table 1:** List of useful drugs for targeting desmoplastic reaction-mediators.

Drug	Refs	Effects
All trans-retinoic acid (ATRA)	[100,101,102]	ATRA inhibits the activation of stellate cells.
Pirfenidone	[103,104,105,106,107,108,109,110,111,112,113,114,115]	Inhibits collagen fibrils assembly; downregulates the intercellular adhesion molecule-1 (ICAM1); decreases the transformation grow factor beta (TGFβ) at the translational level; down-regulates the pro-fibrotic hedgehog signaling pathway; decreases fibroblast proliferation; blocks myofibroblast differentiation; suppresses tumor necrosis factor alpha (TNFα); decreases cell migration-inducing and hyaluronan-binding proteins.
Candesartan	[116]	Angiotensin II receptor inhibitor, which consequently leads to the reduction in stellate cell proliferation.
Olmesartan	[29]	Angiotensin II receptor inhibitor, which consequently leads to the reduction in stellate cell proliferation.
Saridegib (IPI-926)	[98,117,118]	Hedgehog signaling inhibition.
Vismodegib	[119]	Hedgehog signaling inhibition.
4-methyl umbelliferone (4MU)	[85,86,120]	Inhibition of hyaluronan synthase, decreases hyaluronan synthesis; Synergistic activity with gemcitabine.
Curcumin	[121,122,123,124]	Inhibits activation of stellate cells.
L49H37 a curcumin synthetic analog	[125]	Stellate cell inhibitor.
Rhein (natural anthraquinone derivative)	[126,127]	Anti-fibrotic action in PDAC. Reduces collagen I and fibronectin.
Resveratrol	[128]	Impedes stellate cell activation by downregulating miRNA 21. This miRNA is also a participant in gemcitabine resistance.
Emodin	[129,130,131,132]	Emodin has a wide spectrum of activities related with anti-cancer effects and anti-fibrotic actions.
Ellagic acid	[133]	Inhibits the activation and proliferation of stellate cells.
Imatinib	[134,135,136,137,138,139,140,141]	Imatinib is anti-fibrotic in pulmonary-induced fibrosis by bleomycin. It is also anti-fibrotic in breast cancer and the liver. However, in a clinical trial of imatinib associated with gemcitabine it did not show any benefits.
Metformin	[142,143]	Suppresses desmoplasia by activating AMPK and enhances gemcitabine chemosensitivity.
Halofuginone	[144]	Halofuginone is an analog of quinazolinone that shows strong anti-fibrotic properties in an experimental PDAC model. It inhibits the activation of stellate cells.
Pegylated recombinant human hyaluronidase	[145]	Acts by enzymatic degradation of hyaluronate. This device can incorporate chemo drugs including checkpoint inhibitors. Research is ongoing.
Fasudil priming before chemotherapy	[146,147,148,149,150]	Fasudil is a Rho kinase inhibitor. Administered before chemotherapy it decreased stromal density allowing a better level of drug at the tumor.
Pentoxiphyllin	[151,152,153]	Pentoxiphyllin is a reducer of blood viscosity and cytokine production, including TNFα, IL-6, and IL-8 with anti-inflammatory effects and with clear anti-fibrotic effects.
Dasatinib	[154]	Dasatinib decreased pancreatic fibrosis in an experimental model of pancreatitis.
